# Clustering of Pediatric Brain Tumors in Texas, 2000–2017

**DOI:** 10.3390/toxics11040351

**Published:** 2023-04-08

**Authors:** Thanh T. Hoang, Omar Rosales, Elyse Burgess, Philip J. Lupo, Michael E. Scheurer, Abiodun O. Oluyomi

**Affiliations:** 1 Department of Pediatrics, Division of Hematology-Oncology, Baylor College of Medicine, Houston, TX 77030, USA; thanh.hoang@bcm.edu (T.T.H.); philip.lupo@bcm.edu (P.J.L.); scheurer@bcm.edu (M.E.S.); 2 Dan L. Duncan Comprehensive Cancer Center, Baylor College of Medicine, Houston, TX 77030, USA; 3 Cancer and Hematology Center, Texas Children’s Hospital, Houston, TX 77030, USA; 4 Department of Medicine, Epidemiology and Population Sciences Section, Baylor College of Medicine, One Baylor Plaza, Houston, TX 77030, USA; omar.rosales@bcm.edu (O.R.); elyse.burgess@bcm.edu (E.B.)

**Keywords:** childhood brain tumors, hot spot analysis, cluster analysis, central nervous system tumors

## Abstract

Risk factors for pediatric brain tumors are largely unknown. Identifying spatial clusters of these rare tumors on the basis of residential address may provide insights into childhood socio-environmental factors that increase susceptibility. From 2000–2017, the Texas Cancer Registry recorded 4305 primary brain tumors diagnosed among children (≤19 years old). We performed a spatial analysis in SaTScan to identify neighborhoods (census tracts) where the observed number of pediatric brain tumors was higher than expected. Within each census tract, the number of pediatric brain tumors was summed on the basis of residential address at diagnosis. The population estimate from the 2007–2011 American Community Survey of 0- to 19-year-olds was used as the at-risk population. *p*-values were calculated using Monte Carlo hypothesis testing. The age-standardized rate was 54.3 per 1,000,000. SaTScan identified twenty clusters, of which two were statistically significant (*p* < 0.05). Some of the clusters identified in Texas spatially implicated potential sources of environmental risk factors (e.g., proximity to petroleum production processes) to explore in future research. This work provides hypothesis-generating data for further investigations of spatially relevant risk factors of pediatric brain tumors in Texas.

## 1. Introduction

Pediatric brain tumors are the most common solid tumor in children ≤19 years old. Children who develop these tumors experience substantial morbidity and high mortality rates [1,2]. Aside from postnatal exposure to ionizing radiation [3], risk factors related to pediatric brain tumors are largely unestablished.

People are exposed to a mixture of chemicals daily. Some of the variations in chemical exposures can be attributed to nearby geospatial features (e.g., living near agricultural land, major roadways, industrial facilities, etc.) [4]. Spatial-temporal variation of exposure to environmental factors may result in clusters of cancer cases. There is consistent evidence that children with leukemia may aggregate in space and time based on the address at diagnosis [5]. Conversely, it is inconclusive whether pediatric brain tumors cluster in a similar manner. Spatial clustering analyses of pediatric brain tumors have been conducted in the United States and Europe. A systematic review identified 16 publications that have conducted space-time clustering in relation to childhood brain tumors [5]. Of these, four reported an aggregation of any childhood brain tumors. Two studies conducted in Florida used the address at diagnosis and reported a cluster encompassing the Miami–Dade area [6,7]. The other two studies were conducted in Yorkshire, United Kingdom, using both addresses at birth and diagnosis [8] and in Great Britain using the address at birth [9]. Both studies reported clusters, but neither specified the geographical areas that represented the clusters.

Clustering analyses of pediatric brain tumors conducted in the United States have been conducted in a few states (i.e., California, Florida, Georgia, and New Jersey) and have used different geographical units, including counties [10,11], Zip Code Tabulation Areas (ZCTA—approximates U.S. postal service zip codes) [7,12], and census tracts [6]. Census tracts provide greater geospatial granularity than counties and ZCTAs, especially in dense urban areas.

Texas is the second most populous state in the United States [13] and the second in the number of newly diagnosed pediatric cancer cases each year [14]. Texas is also geographically diverse in terms of its urbanicity and economic industries (e.g., agriculture, oil and gas production). No studies have conducted a clustering analysis within Texas. Therefore, to generate hypotheses related to the etiology of pediatric brain tumors, we conducted a clustering analysis with the census tract as the unit of analysis in the state of Texas for the period 2000–2017. By identifying the areas of the state where incidence rates may be higher than expected, the results could further guide the investigation of potential risk factors for pediatric brain tumors in Texas.

## 2. Materials and Methods

### 2.1. Study Population

The Texas Cancer Registry (TCR) is the statewide, population-based registry that utilizes active and passive surveillance systems to collect cancer cases in Texas. The TCR is one of the largest cancer registries in the United States. TCR collects information required by the North American Association of Central Cancer Registries (NAACCR) and National Cancer Institute’s Surveillance, Epidemiology and End Results (SEER) Program, including the types of cancer diagnosed and their locations within the body on the basis of ICD-O-3 codes. For children ≤19 years old with a recorded tumor diagnosed, the ICD-O-3 histology, primary site, and behavior codes were used to classify the tumor based on the International Classification of Childhood Cancer (ICCC), 3rd edition [15]. From 2000–2017, 4305 children were diagnosed with a primary malignant brain tumor (i.e., categorized as group III on the basis of the ICCC with an ICD-O-3 behavior code of 3).

### 2.2. Cluster Analysis

Using SaTScan software [16], we conducted a cluster analysis that used the census tract as the unit of analysis. We retrieved the census tract geographic file from the US Census Bureau TIGER database that matched the 2007–2011 American Community Survey (ACS) estimates. To obtain the total population at risk within each census tract, we used the U.S. Census population estimates based on the midpoint of our study period (i.e., 2009), which was the American Community Survey’s 5-year population estimate of 0- to 19-year-olds for 2007–2011. The Texas Department of State Health Services Center for Health Statistics geocoded each patient’s residential address at diagnosis, making it possible to sum the number of pediatric brain tumors inside each census tract. Because of the nature of the data, we formatted the analysis as Poisson distribution [17], representing the brain tumor diagnosis as events. SaTScan imposes a moving window over a geographical area. Census tracts are represented by their centroids and are included in windows that contain their centroids. Circular windows were set, such that SaTScan could run an infinite number of circles around each centroid until the window reached a maximum radius of 50% of the population at risk was included. SaTScan tested whether the incidence of pediatric brain tumors in census tracts within a window was greater than the incidence in census tracts outside the window by calculating the likelihood function. SaTScan uses Monte Carlo hypothesis testing to calculate the *p*-value by comparing the rank of the maximum likelihood from our data with the maximum likelihoods from randomly generated data sets [16,18].

## 3. Results

The age-adjusted incidence rate for pediatric brain tumors in Texas over our study period was 54.3 per 1,000,000 children. The rates were calculated using the 2000 US Standard Population for age standardization. The median age of cases was 7 years. Cases were more likely to be male (53%) (Table 1). By race/ethnicity, 48% were non-Hispanic white, 37% were Hispanic, 11% were non-Hispanic Black, and 4% were Other or Unknown. Six cases had missing latitude or longitude information, leaving 4299 for the cluster analysis.

**Figure 1:**
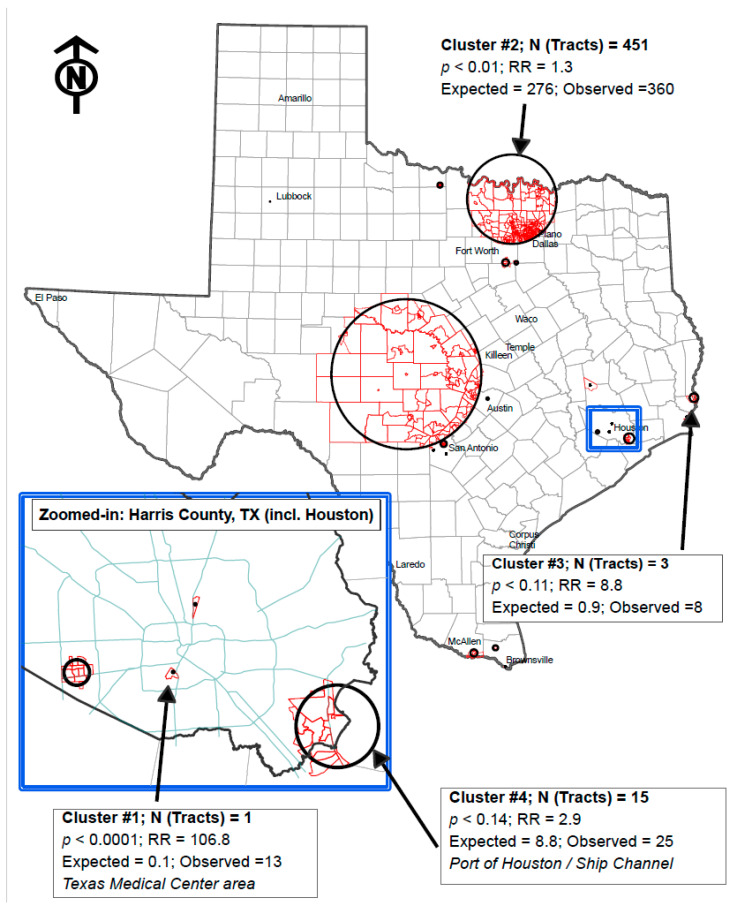
Clusters of pediatric brain tumors in Texas, 2000–2017. Black arrows indicate clusters with a *p* < 0.15.

SaTScan identified 20 clusters, of which two were statistically significant at *p* < 0.05 (Figure 1, Table 2). The most significant cluster (*p* < 0.001) was identified in a census tract encompassing the Texas Medical Center. The Texas Medical Center is home to two renowned cancer centers that treat childhood cancers (i.e., Texas Children’s Hospital and MD Anderson Cancer Center). The second significant cluster spatially encompassed 451 census tracts in the larger Dallas–Fort Worth metropolitan area (most located in Montague, Cooke, Grayson, Wise, Denton, and Collin County) (*p* = 0.01). Two clusters had a *p*-value greater than 0.05 and less than 0.15. One of these clusters contained three census tracts in Orange County, Texas (*p* = 0.11). The other contained 15 census tracts along part of the Houston Ship Channel (*p* = 0.14). While the remaining 16 clusters had a *p*-value greater than 0.15, SaTScan still identified them as non-random aggregations of pediatric brain tumors.

## 4. Discussion

Overall, we identified 20 clusters of childhood brain tumors across Texas, of which 2 were statistically significant. Below, we hypothesize as to what these geospatial clusters may reflect.

The most significant hotspot was identified around the Texas Medical Center, which is home to two world-renowned pediatric cancer treatment centers. Physicians across the United States and the world may refer their patients to these cancer treatment centers. We speculate that this cluster reflects families temporarily relocating to the Texas Medical Center to receive care. While a non-significant cluster was identified a few miles outside of McAllen, Texas (cluster #18, Figure S1), this cluster was near the Vannie Cook Children’s Clinic, which is the comprehensive pediatric cancer and hematology center in South Texas. We also hypothesize that this cluster might reflect the referrals of patients to Vannie Cook by physicians in South Texas or Mexico who might temporarily relocate to this area for treatment. Further research is needed to understand what address is being collected at the time of diagnosis and if changes in the data collection or reporting procedures may be necessary.

The second most significant cluster was geospatially the second largest, contained the largest number of census tracts, and included a large portion of North Texas. There are several possible hypotheses that could contribute to this cluster. First, the Dallas–Fort Worth Metropolitan Area experienced substantial population growth in the northern suburbs during this study period [19]. Although we used the census population estimates based on the midpoint of our study period (i.e., 2009) to represent the population at risk, it is possible that some census tracts might have experienced faster growth, which we were unable to account for. Second, part of the large cluster overlaps with the Barnett Shale, an area that has large reserves of natural gas and some permitted gas drilling. Extracting these resources requires a multistep process that includes pad preparation, drilling, hydraulic fracking, and gas production. Each process releases or injects different compounds into the environment [20]. Pad preparation, drilling, and oil and gas production release air pollution [20]. Hydraulic fracking injects fracking fluid (a mixture of water, sand, and chemicals) into the well site to create cracks in the deep rocks to better access the gas or oil, which may contaminate sources of drinking water [20]. While not conclusive, there is evidence that living near oil and gas operations is associated with adverse health effects, including poorer reproductive outcomes and respiratory conditions [21]. Living near hydraulic fracking sites has also been reported to elevate the risk of childhood leukemia [22,23], but the literature regarding pediatric brain tumors is sparse. One study reported an elevated risk of pediatric brain tumors, but the risk was higher in counties with the fewest number of wells [24]. Third, the southern part of the cluster is near the Dallas–Fort Worth airport—one of the busiest airports in the United States. Airplanes emit various air pollutants, including ultrafine particles and volatile organic compounds. Concentrations of air pollutants have been reported to be higher around airports and downstream of landing and takeoff [25]. One study reported that the incidence of childhood leukemia in Texas tended to be higher in census tracts near airports [26], but no study was found investigating proximity to airports and the risk of pediatric brain tumors. The literature on air pollutants and pediatric brain tumors is inconclusive, but some positive associations have been reported [3].

While not significant, the third and fourth clusters were geospatially near ports where petroleum and petroleum products are imported and exported or near oil and gas refineries. The cluster in Orange County was geographically located between Port Arthur, Texas, in the southwest and Lake Charles, Louisiana, in the east. These two cities are home to two of the largest petroleum refineries in the United States and have ports for importing and exporting oil and gas products. The fourth cluster included part of the Houston Ship Channel, one of the world’s busiest ports, and was located near oil and gas refineries. Another non-significant hotspot (cluster #13, Figure S1) was also identified in Port Arthur. Together, these clusters suggest that being exposed to pollutants from these industries may increase susceptibility to pediatric brain tumors, warranting further research to test this hypothesis.

Our clustering analysis can only identify areas where the number of observed cases of childhood brain tumors is greater than expected. Because we did not conduct any statistical inference tests between specific exposures and the risk of childhood brain tumors, we cannot draw any definitive conclusions about why these clusters were identified. Further research is needed to test the hypotheses proposed above between potential sources of pollutants and the risk of childhood brain tumors. The proposed sources of pollutants discussed above emit a mixture of pollutants (e.g., metals, particulate matter, and benzene [20,25,27]) that can be inhaled or ingested and cross the blood–brain barrier [28]. Several of these pollutants have been linked to adverse neurodevelopmental outcomes in children [28] and adult brain tumors [29].

Pediatric brain tumors have over 100 histological subtypes and thus are more heterogeneous than childhood leukemia. In the systematic review, 10 of the 16 publications did not report a cluster with pediatric brain tumors [5]. Given the heterogeneity of childhood brain tumors, it is possible that childhood brain tumors may cluster by histological subtypes. Eight studies reported in the literature have conducted analyses by histological type. Two (one in North West England and the other in Murcia, Spain) reported a cluster with astrocytoma [30,31] and one with primitive neuro-ectodermal tumors (PNET) in Yorkshire, United Kingdom [8]. Another study published after the systematic review reported a cluster of astrocytomas in California [12]. Three studies reported conducted geospatial analyses with medulloblastoma or ependymoma [5], but no cluster was identified. It may be challenging to identify clusters of medulloblastoma and ependymoma given that there are four and nine molecular subtypes, respectively. The molecular characterization of medulloblastoma was recognized by the World Health Organization in 2016 [32] for ependymoma in 2021 [33]. These molecular characterizations were not collected in the Texas Cancer Registry during this study period. Given our decision to conduct the spatial clustering analysis using census tract to identify areas with more granularity, we did not conduct analyses by histological subtype due to the smaller sample size. As the Texas Cancer Registry continues to collect data on cases diagnosed in the state, future studies may be able to conduct clustering analyses by specific subtypes of pediatric brain tumors.

This study has some limitations. Because we did not have the address at birth, we were unable to repeat the geospatial analyses at this timepoint and compare clusters. While we cannot rule out the prenatal period as a critical window of exposure, there is some evidence that exposures based on addresses at birth and diagnosis are similar [34,35], so these results may also apply to sources of exposure during pregnancy. In our analyses, we used Kulldorff’s circular scan, which may not capture non-circular clusters. Strengths of this study include having latitude and longitudinal data of cases, its large sample size and representation of almost all cases of childhood brain tumors diagnosed in the state, the geographical diversity based on urbanicity and economic industries, and utilizing census tracts as the geographical unit.

In conclusion, this is the first geospatial clustering analysis of childhood brain tumors diagnosed in Texas, in which several clusters were identified. Some of these clusters geospatially overlap in areas with known sources of environmental pollutants. While this study cannot draw conclusions about these pollutants and the risk of childhood brain tumors, future research is warranted to test the hypotheses generated from our results for both exposures during pregnancy and early childhood.

## Figures and Tables

**Table 1:** Characteristics of children diagnosed with pediatric brain tumor, Texas, 2000–2017.

	*n* = 4035
Age (yrs)	*n* (%)
<1	242 (5.6)
1–4	1235 (28.7)
5–9	1235 (28.7)
10–14	893 (20.7)
15–19	700 (16.3)
Sex	
Male	2294 (53.3)
Female	2011 (46.7)
Race/ethnicity	
Non-Hispanic White	2078 (48.3)
Hispanic	1608 (37.4)
Non-Hispanic Black	452 (10.5)
Other/Unknown	167 (3.9)
Histological subtype	
Astrocytoma	2081
Medulloblastoma	555
Ependymoma	371

**Table 2:** Summary of clusters of pediatric brain tumor, Texas, 2000–2017.

Cluster	Population	Observed	Expected	Relative Risk	*p*-Value	No. of CTs in Cluster
1	291	13	0.1	106.8	0.000	1
2	657,049	360	276	1.3	0.01	451
3	2176	8	0.9	8.8	0.11	3
4	20,931	25	8.8	2.9	0.14	15
5	4186	10	1.8	5.7	0.28	3
6	11,646	17	4.9	3.5	0.29	7
7	811	5	0.3	14.7	0.37	1
8	145	3	0.1	49.3	0.38	1
9	8513	14	3.6	3.9	0.40	6
10	13,461	17	5.7	3.0	0.80	13
11	5235	10	2.2	4.6	0.81	4
12	24,885	25	10.5	2.4	0.83	22
13	250	3	0.1	28.6	0.86	1
14	171,893	105	72.2	1.5	0.95	143
15	1289	5	0.5	9.2	0.95	1
16	719	4	0.3	13.3	0.96	1
17	756	4	0.3	12.6	0.98	1
18	20,528	21	8.6	2.4	0.98	11
19	17,968	19	7.5	2.5	0.99	12
20	858	4	0.4	11.1	0.99	1

CTs—census tracts.

## Data Availability

Restrictions apply to the availability of these data. Data were obtained from the Texas Department of State Health Services, and, to protect privacy and confidentiality, cannot be made available to third parties. Investigators may apply to the Texas Cancer Registry to access the underlying data.

## Supplementary Materials

The following are available online at https://www.mdpi.com/article/10.3390/toxics11040351/s1, Figure S1: Map of pediatric brain tumor clusters labeled, Texas, 2000–2017.

## References

[1] Curtin S.C., Minino A.M., Anderson R.N. (2016). Declines in Cancer Death Rates Among Children and Adolescents in the United States, 1999–2014.

[2] Oyefiade A., Paltin I., De Luca C.R., Hardy K.K., Grosshans D.R., Chintagumpala M., Mabbott D.J., Kahalley L.S. (2021). Cognitive Risk in Survivors of Pediatric Brain Tumors. J. Clin. Oncol..

[3] Hoang T.T., Whitcomb E., Reardon E.E., Spector L.G., Lupo P.J., Scheurer M.E., Williams L.A. (2022). Environmental Risk Factors for Childhood Central Nervous System Tumors: An Umbrella Review. Curr. Epidemiol. Rep..

[4] Eccles K.M., Karmaus A.L., Kleinstreuer N.C., Parham F., Rider C.V., Wambaugh J.F., Messier K.P. (2023). A geospatial modeling approach to quantifying the risk of exposure to environmental chemical mixtures via a common molecular target. Sci. Total Environ..

[5] Kreis C., Doessegger E., Lupatsch J.E., Spycher B.D. (2019). Space-time clustering of childhood cancers: A systematic review and pooled analysis. Eur. J. Epidemiol..

[6] Kearney G. (2008). A procedure for detecting childhood cancer clusters near hazardous waste sites in Florida. J. Environ. Health.

[7] Amin R., Hendryx M., Shull M., Bohnert A. (2014). A Cluster Analysis of Pediatric Cancer Incidence Rates in Florida: 2000–2010. Stat. Public Policy.

[8] McNally R.J., James P.W., Picton S.V., McKinney P.A., van Laar M., Feltbower R.G. (2012). Space-time clustering of childhood central nervous system tumours in Yorkshire, UK. BMC Cancer.

[9] McNally R.J., Bithell J.F., Vincent T.J., Murphy M.F. (2009). Space-time clustering of childhood cancer around the residence at birth. Int. J. Cancer.

[10] Schneider D., Greenberg M.R., Donaldson M.H., Choi D. (1993). Cancer clusters: The importance of monitoring multiple geographic scales. Soc. Sci. Med..

[11] Kanu F.A., Wagner Robb S., Corriero R. (2015). Childhood cancer incidence in Georgia: Descriptive epidemiology, geographic trends, and disparities in insurance coverage and health care access. J. Ga. Public Health Assoc..

[12] Francis S.S., Enders C., Hyde R., Gao X., Wang R., Ma X., Wiemels J.L., Selvin S., Metayer C. (2020). Spatial-Temporal Cluster Analysis of Childhood Cancer in California. Epidemiology.

[13] United States Census Bureau (2023). Annual Estimates of the Resident Population: April 1, 2010 to July 1, 2019.

[14] Siegel D.A., Li J., Henley S.J., Wilson R.J., Lunsford N.B., Tai E., Van Dyne E.A. (2018). Geographic Variation in Pediatric Cancer Incidence—United States, 2003–2014. MMWR Morb. Mortal. Wkly. Rep..

[15] Steliarova-Foucher E., Stiller C., Lacour B., Kaatsch P. (2005). International Classification of Childhood Cancer, third edition. Cancer.

[16] Kulldorff M. (2022). SaTScan User Guide v10.01. https://www.satscan.org/cgi-bin/satscan/register.pl/SaTScan_Users_Guide.pdf?todo=process_userguide_download.

[17] Kulldorff M. (1997). A Spatial Scan Statistic. Commun. Stat. Theory Methods.

[18] Dwass M. (1957). Modified randomization tests for nonparametric hypotheses. Ann. Math. Stat..

[19] Kotkin J., Clark C. (2021). Big D is a Big Deal: Dallas-Fort Worth is becoming the de facto capital of America’s Heartland. City J. Summer.

[20] Wollin K.M., Damm G., Foth H., Freyberger A., Gebel T., Mangerich A., Gundert-Remy U., Partosch F., Rohl C., Schupp T. (2020). Critical evaluation of human health risks due to hydraulic fracturing in natural gas and petroleum production. Arch. Toxicol..

[21] Bamber A.M., Hasanali S.H., Nair A.S., Watkins S.M., Vigil D.I., Van Dyke M., McMullin T.S., Richardson K. (2019). A Systematic Review of the Epidemiologic Literature Assessing Health Outcomes in Populations Living near Oil and Natural Gas Operations: Study Quality and Future Recommendations. Int. J. Environ. Res. Public Health.

[22] McKenzie L.M., Allshouse W.B., Byers T.E., Bedrick E.J., Serdar B., Adgate J.L. (2017). Childhood hematologic cancer and residential proximity to oil and gas development. PLoS ONE.

[23] Clark C.J., Johnson N.P., Soriano M., Warren J.L., Sorrentino K.M., Kadan-Lottick N.S., Saiers J.E., Ma X., Deziel N.C. (2022). Unconventional Oil and Gas Development Exposure and Risk of Childhood Acute Lymphoblastic Leukemia: A Case-Control Study in Pennsylvania, 2009–2017. Environ. Health Perspect..

[24] Fryzek J., Pastula S., Jiang X., Garabrant D.H. (2013). Childhood cancer incidence in Pennsylvania counties in relation to living in counties with hydraulic fracturing sites. J. Occup. Environ. Med..

[25] Bendtsen K.M., Bengtsen E., Saber A.T., Vogel U. (2021). A review of health effects associated with exposure to jet engine emissions in and around airports. Environ. Health.

[26] Senkayi S.N., Sattler M.L., Rowe N., Chen V.C.P. (2014). Investigation of an association between childhood leukemia incidences and airports in Texas. Atmos. Pollut. Res..

[27] Wallace H.W., Sanchez N.P., Flynn J.H., Erickson M.H., Lefer B.L., Griffin R.J. (2018). Source apportionment of particulate matter and trace gases near a major refinery near the Houston Ship Channel. Atmos. Environ..

[28] Webb E., Moon J., Dyrszka L., Rodriguez B., Cox C., Patisaul H., Bushkin S., London E. (2018). Neurodevelopmental and neurological effects of chemicals associated with unconventional oil and natural gas operations and their potential effects on infants and children. Rev. Environ. Health.

[29] Connelly J.M., Malkin M.G. (2007). Environmental risk factors for brain tumors. Curr. Neurol. Neurosci. Rep..

[30] McNally R.J., Cairns D.P., Eden O.B., Alexander F.E., Taylor G.M., Kelsey A.M., Birch J.M. (2002). An infectious aetiology for childhood brain tumours? Evidence from space-time clustering and seasonality analyses. Br. J. Cancer.

[31] Ortega-Garcia J.A., Lopez-Hernandez F.A., Fuster-Soler J.L., Martinez-Lage J.F. (2011). Space-time clustering in childhood nervous system tumors in the Region of Murcia, Spain, 1998-2009. Childs Nerv. Syst..

[32] Louis D.N., Perry A., Reifenberger G., von Deimling A., Figarella-Branger D., Cavenee W.K., Ohgaki H., Wiestler O.D., Kleihues P., Ellison D.W. (2016). The 2016 World Health Organization Classification of Tumors of the Central Nervous System: A summary. Acta Neuropathol..

[33] Louis D.N., Perry A., Wesseling P., Brat D.J., Cree I.A., Figarella-Branger D., Hawkins C., Ng H.K., Pfister S.M., Reifenberger G. (2021). The 2021 WHO Classification of Tumors of the Central Nervous System: A summary. Neuro-Oncology.

[34] Danysh H.E., Mitchell L.E., Zhang K., Scheurer M.E., Lupo P.J. (2017). Differences in environmental exposure assignment due to residential mobility among children with a central nervous system tumor: Texas, 1995–2009. J. Expo. Sci. Environ. Epidemiol..

[35] Ling C., Heck J.E., Cockburn M., Liew Z., Marcotte E., Ritz B. (2019). Residential mobility in early childhood and the impact on misclassification in pesticide exposures. Environ. Res..

