# Supplementary figures and images for: Clustering of Pediatric Brain Tumors in Texas, 2000–2017
Source: Toxics. 2023 Apr 8;11(4):351. doi: 10.3390/toxics11040351 (PMC10146099; doi:10.3390/toxics11040351)

**Figure S1. Map of pediatric brain tumor clusters labeled, Texas, 2000-2017**

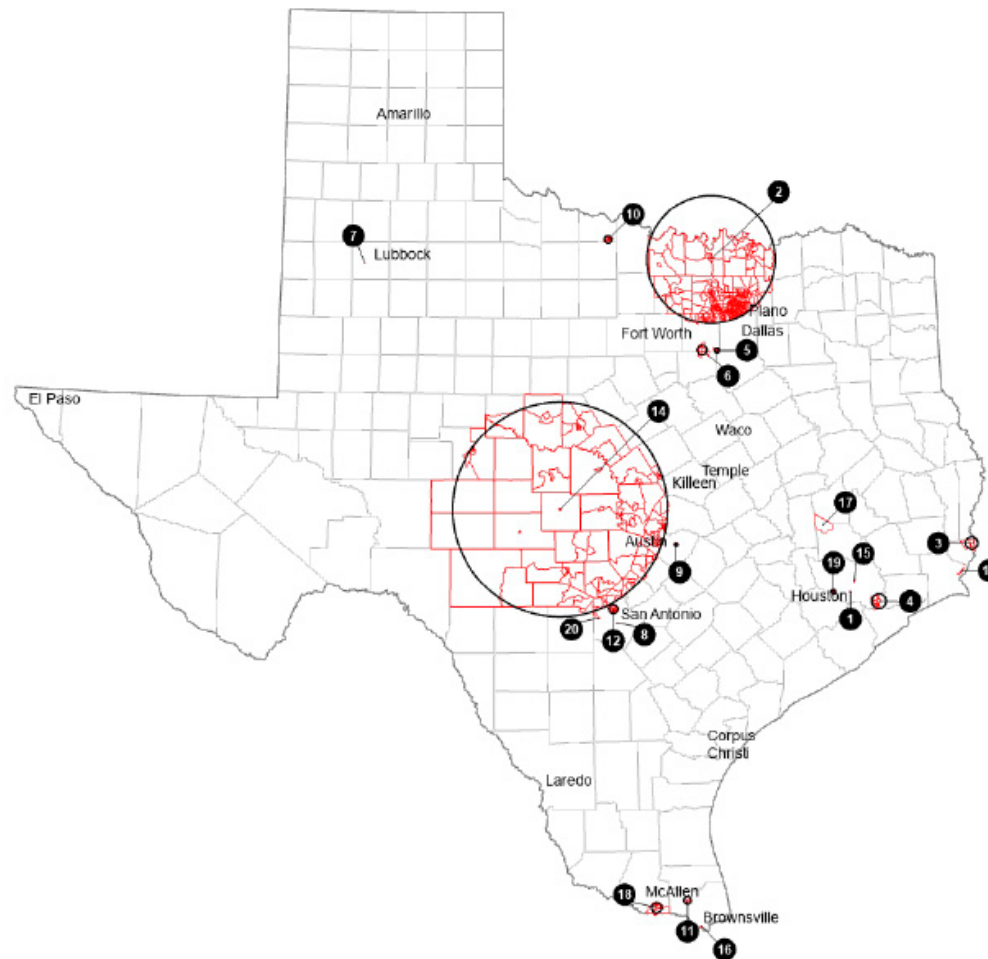

Supplement: Supplementary file 1 [file toxics-11-00351-s001.zip › toxics-2280764-supplementary.pdf]
